# Diversity of Phage-Displayed Libraries of Peptides during Panning and Amplification

**DOI:** 10.3390/molecules16021776

**Published:** 2011-02-21

**Authors:** Ratmir Derda, Sindy K.Y. Tang, S. Cory Li, Simon Ng, Wadim Matochko, Mohammad R. Jafari

**Affiliations:** 1 Department of Chemistry, University of Alberta, Edmonton, AB T6G 2G2, Canada; 2 School of Engineering and Applied Sciences, Harvard University, Cambridge, MA 02138, USA; 3 Department of Bioengineering, Massachusetts Institute of Technology, Cambridge, MA 02139, USA

**Keywords:** phage display, diversity, competition, amplification, fast-growing clones

## Abstract

The amplification of phage-displayed libraries is an essential step in the selection of ligands from these libraries. The amplification of libraries, however, decreases their diversity and limits the number of binding clones that a screen can identify. While this decrease might not be a problem for screens against targets with a single binding site (e.g., proteins), it can severely hinder the identification of useful ligands for targets with multiple binding sites (e.g., cells). This review aims to characterize the loss in the diversity of libraries during amplification. Analysis of the peptide sequences obtained in several hundred screens of peptide libraries shows explicitly that there is a significant decrease in library diversity that occurs during the amplification of phage in bacteria. This loss during amplification is not unique to specific libraries: it is observed in many of the phage display systems we have surveyed. The loss in library diversity originates from competition among phage clones in a common pool of bacteria. Based on growth data from the literature and models of phage growth, we show that this competition originates from growth rate differences of only a few percent for different phage clones. We summarize the findings using a simple two-dimensional “phage phase diagram”, which describes how the collapse of libraries, due to panning and amplification, leads to the identification of only a subset of the available ligands. This review also highlights techniques that allow elimination of amplification-induced losses of diversity, and how these techniques can be used to improve phage-display selection and enable the identification of novel ligands.

## 1. Introduction—Motivation for Writing This Review

Receptor-ligand interactions are the basis for most biological processes. The discovery of ligands that bind a specific target is the basis for the development of pharmaceuticals, biomaterials, and diagnostic tools. There are, in general, two strategies for the development of ligands for a new target: (1) rational design and (2) selection from libraries of random molecules. Within the second strategy, phage display is a widely-used method that allows for the identification of useful ligands from a library of 10^9^ random polypeptides [1,2,3,4]. The expression of peptides on the coat proteins of bacteriophage physically links the peptide to the information carrier (DNA) inside phage that contains all the instructions for the synthesis of the expressed peptide [5,6,7]. Unlike conventional chemical libraries [8,9,10,11,12,13], each member of the phage-display library, even if present as a single “molecule” (*i.e.,* one phage particle), can be amplified to an amount sufficient for detection or assay. Phage display has been used to discover ligands for a wide range of targets, including proteins, cells and tissues, and even inorganic materials (for reviews see [6,14,15,16,17,18,19,20,21]). The number of discovered ligands, however, is often lower than expected from a library of 10^9^ diverse peptides. For example, some targets—such as cells, tissues and organs—have many binding sites, but multiple groups reported a convergence to <5 ligands after rounds of panning and amplification. Factors other than the binding affinity between ligands and the target must also contribute to the convergence of the library to the identified ligands.

The amplification of libraries, which is an essential step in phage display selection, has been shown to decrease the diversity of libraries [22,23,24,25]. Literature summarizing the effects of amplification is rare. The motivation of this review, therefore, is to organize the findings from the phage display literature and to show explicit evidence that the amplification of libraries leads to a loss of useful binding ligands. The elimination of the processes that lead to the undesired loss of diversity during amplification enables the identification of a much broader repertoire of binding ligands, including the identification of multiple ligands for targets with multiple binding sites (e.g., cells, tissues). We describe two approaches that have been used to bypass this unwanted loss of diversity: (1) selection without amplification; (2) amplification in isolated compartments. We also discuss approaches to characterize the loss of diversity in current phage display screens: (1) deep-sequencing of phage libraries; (2) bio-informatic analyses of library diversities; (3) databases of phage-display screens.

This review focuses on libraries based on functional filamentous phage, which is used to display short peptides. The loss of diversity during amplification also occurs in related techniques based on phagemid-display [26,27] which is used to display natural [28,29] or synthetic [3,30,31] antibody fragments and other full-length proteins [32,33], as well as displays with other types of phage (T4, λ). Competition during amplification is not unique to phage; it also occurs in other display systems. We will outline them briefly in the last section of this review.

## 2. The Problem: Panning *vs*. Rate of Amplification

**Figure 1:**
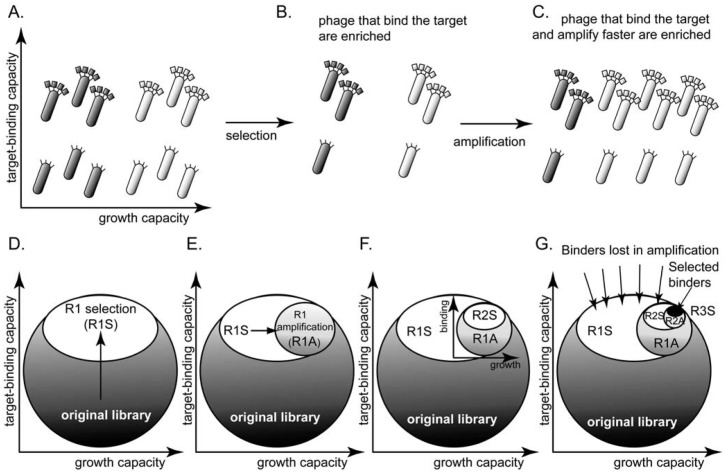
**(A)** A library of phage-displayed peptides contains clones that bind to a target better than other clones and clones that amplify faster than other clones. These characteristics are largely independent. **(B)** A round of panning enriches the phage clones that bind to the target. **(C)** A round of amplification enriches for the clones that amplify faster. Presenting the library as a circle in the (binding vs. growth)-phase diagram allows the description of **(D)** selection (R1S) as a collapse to the upper part of the circle and **(E)** amplification (R1A) as further collapse to the right part of the phase diagram. **(F-G)** The decrease in diversity in subsequent rounds of screening and amplification is identical to that in **(D-E)**; it leads to a collapse of the sub-population to the upper-right portion. **(G)** After three rounds of selection, the screen identifies binding ligands. The number of identified ligands, however, is much smaller than the number of binders that were originally present in the library.

Selection from phage display libraries is driven by two processes (Figure 1): (1) the panning step enriches for clones that bind to the desired target or any other physical moieties present during the panning step, such as walls of the vessels [34], *etc.* (reviewed in [35]) (Figure 1A,B). (2) The amplification step—infection of bacteria by a single phage particle and the secretion of ~1,000 copies of phage—enriches clones that have an advantage during any of the amplification steps [24,36,37,38,39] (Figure 1B,C). 

If processes (1) and (2) are largely independent, the phage library can be represented using a two-dimensional “phage phase diagram” (Figure 1D): the top of the diagram contains the library members that bind to the target with the strongest affinity, and right of the diagram contains the library members that have the highest growth advantage. Figure 1D-G describe a hypothetical selection process: panning collapses the circular shape to the top, “strong-binding” part of the library (Figure 1D). Amplification collapses the ellipse horizontally towards the right of the diagram because (binding) clones that amplify faster out-compete other (binding) clones that amplify slower (Figure 1E). Subsequent rounds of panning and amplification—described by a series of vertical and horizontal collapses—yield a small number of ligands that bind strongly to the target. The number of these ligands, however, is much smaller than the number of all binders in the library (Figure 1E). 

The diagram described in Figure 1D is a qualitative description accounting for the two independent selection pressures caused by panning and amplification. It makes predictions that cannot be inferred from models based solely on binding events during panning [40,41,42]: (1) selection identifies a sub-population of the binding ligands that have high growth rate only. (2) Selection pressures are orthogonal (independent): increasing one does not eliminate the effect of the other. (3) The loss of diversity during amplification can be minimized only by reducing the number of amplification steps, or by eliminating differences in growth rate of different clones. Indeed, it is known that 3-4 rounds of selection provide ligands of the highest diversity. Multiple rounds of selection (>4) improve binding ability only partially, but they are known to in fact reduce sequence diversity

This phage phase diagram is useful to visualize qualitatively the loss of diversity that occurs during selection process (panning + amplification). In the following sections, we present results from phage display screens in the literature to provide examples and analysis on the loss in diversity during amplification. 

## 3. Evidence for Amplification-Induced Convergence Based on Comparison of Sequence Diversity at Every Step of the Selection Process

**Figure 2:**
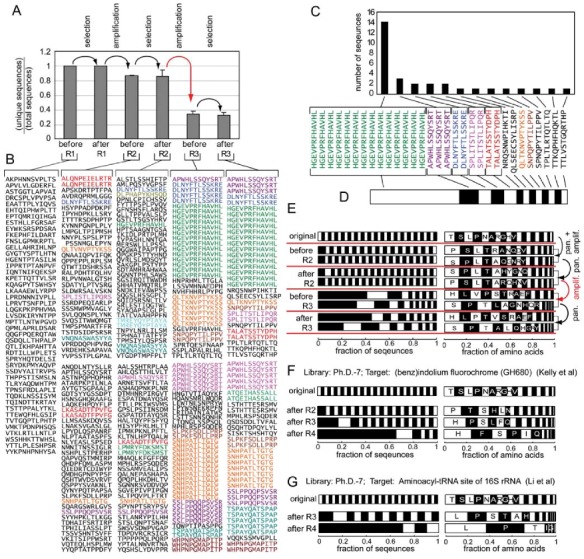
**(A)** We performed panning starting from the Ph.D.-12 phage library using hES cells as the target. Amplification occurred in a standard shaking culture. After each round of panning or amplification, we sequenced 40-50 clones. The plot in **(A)** summarizes the results from two independent experiments. The diversity of the library collapses at each amplification step. **(B)** Sequences from each round; repeating sequences are colored. **(C) **Distribution of the clones in the library can be presented as a stacked bar chart **(D)** in which the width of each black or white bar is proportional to the abundance of the peptide sequence. **(E)** presents the same data as A and B using stacked bars. Two replicates are presented independently. The right set of bars describe the abundance of individual amino acids in the library. **(F)** describes the sequencing results from Kelly *et al.* [46], and Li *et al.* [47]. (A and B are reproduced from Derda *et al.* [23] with permission). The frequencies of amino acids (AA) in E, G, F (and in subsequent Figure 3 and Figure 4) were calculated as (number of times AA encountered) / (total number of AAs in all sequences).

In our previous report, we performed a small-scale investigation by sequencing a small fraction of the library (40-60 clones) after each round of panning and amplification [23]. The panning target—human embryonic stem (hES) cells—is one that has plethora of binding sites (1,000s of receptors on the cell surface). The screen targeted all of the receptors on the surface of hES cell and should have identified a vast number of binding ligands. We observed that the diversity of the final library was low: the results after three rounds were dominated by less than ten peptide sequences (Figure 2A,B). Several of them (APWHLSSQYSRT and HGEVPRFHAVHL) were confirmed by Lan Ma and co-workers to be specific ligands that bind to the surface of primate ES cells or neural stem cells [43,44,45]. It was surprising that the screen converged on a few peptide sequences only. By tracing the loss of diversity in the amplification/selection process, we saw that the diversity of the library decreased abruptly at each amplification step. A single amplification step eliminated ~70% of the phage clones. Some of them—for example LPMRYFDKSMST, TMREYQYPTAYA and VNQNASWASYYA—were present at 2/40 abundance before amplification, and thus were enriched in the second round of the selection (column 3, Figure 2B). These peptides, however, disappeared after amplification, *i.e.*, their abundances dropped from 2/40 to < 1/40 after amplification (column 4, Figure 2B).

We could not find any other examples in the literature that sequenced clones after each round of selection and amplification separately, although there are reports that describe deep sequencing of libraries of RNA aptamers after each round of amplification [48]. These reports also demonstrate that panning and amplification processes impose two independent selection pressures on the library (see discussion below). In the phage display literature, many reports describe sequences of the phage libraries after every round of panning. In order to facilitate comparison of library diversity from different reports in the following sections, we summarize and present the sequence results after each round(s) of panning or amplification as a stacked bar (Figure 2D). Each segment represents a unique sequence. The length of the segment is proportional to the relative abundance of the sequence (Figure 2C). This presentation contains a higher density of information than a simple bar graph and is more space-effective than lists of sequences (compare Figure 2A, B and E). 

We show an example of the charts representing loss of diversity in a library of 7-mers (Ph.D.-7) in a screen for ligands that bind (benz)indolium fluorochrome (GH680) (Figure 2F) [46]; or aminoacyl-tRNA site (A site) of 16S rRNA (Figure 2G) [47]. The collapse of sequence diversity in Figure 2F-G is similar to that in Figure 2E. Both screens converge abruptly to a small number of ligands after a few rounds of the screening, even when the libraries or targets were very different. 

## 4. Evidence for Amplification-Induced Convergence Based on Comparison of the Diversity of Identified Ligands for Targets with One, Few, Or Many Binding Sites

We compare the final diversities of the ligands identified in the screens against diverse targets. In comparing these results, it is useful to categorize the targets according to the expected number of binding ligands. **Category 1:** If the target has one well-defined binding site that recognizes a defined amino acid sequence (e.g., monoclonal antibodies), one should identify a relatively low number of sequences for this target. **Category 2:** If the target has several binding sites (e.g., polyclonal antibodies), or if the binding site is less defined (e.g., proteins with large binding interfaces or inorganic materials), the number of ligands should be greater than that identified for ligands in the category 1. **Category 3:** If the target has 1,000s of binding sites (e.g., cells or organs), there should be a significantly higher number of ligands identified for these targets when compared to both categories 1 and 2.

**Figure 3:**
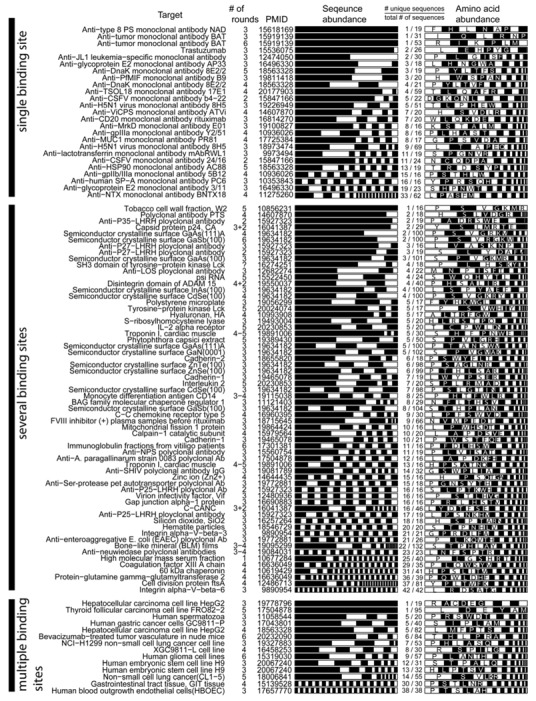
Analysis of the diversity of the Ph.D.-12 phage library after screening against various targets (see Figure 2 for an explanation of the stacked bar representation) from papers that report >15 DNA sequences. The data was extracted from raw MimoBD database [49] using a custom MatLab software. PMID is the PubMed ID of each article.

**Figure 4:**
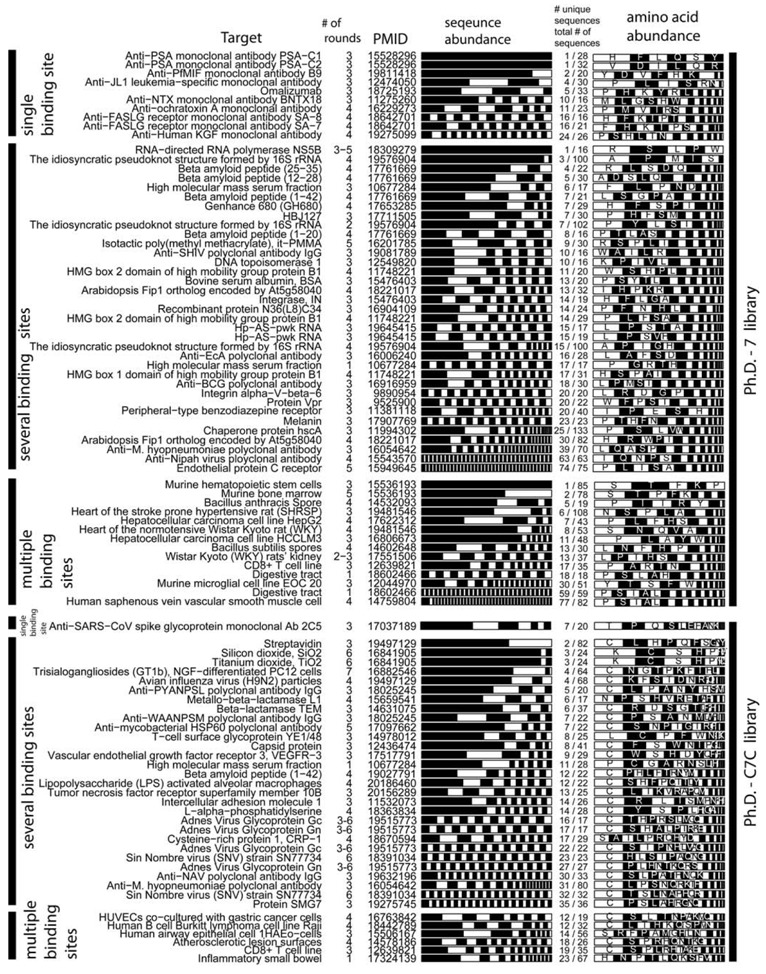
Analysis similar to Figure 3, but for Ph.D.-7 and Ph.D.-C7C libraries.

We selected studies that originated from three types of libraries: Ph.D-12^TM^, Ph.D-7^TM^, and Ph.D-C7C^TM^ library of 12-mers, 7-mers, and cyclic 7-mers, respectively, displayed on protein P-III (New England BioLabs). Figure 3 and Figure 4 used references that contained information about > 15 clones only. We clustered the results according to the target category and sorted them according to the number of unique sequences (column 5, Figure 3 and 4). Figure 3 and Figure 4 contain several useful observations. (1) Within each target category, there are similar abundances of screens that yield one or multiple unique sequences. (2) There is no obvious correlation between the number of binding sites in the target and the number of unique binding clones identified for this target. (3) There is no apparent correlation between the distribution of binding clones (patterns within the stacked bars) and the nature of the target. We observed similar trends when we analyzed other libraries that were used in a large number of screens (e.g., fUSE5-based libraries developed by Smith and co-workers [7]). The results from ~300 screens indicate that the selection against any target always converges to a small number of ligands.

Theoretically, more ligands should be identified for the targets that have more binding sites. In reality, the distribution of diversities is similar for all targets (Figure 3, Figure 4). The convergence to a few binding ligands is unexpected, as a library of 10^9^ peptides should contain 10^2^-10^5^ ligands of similar affinities (see below: “How many binding ligands are lost in the screen?”). The survey of the literature, shown in Figure 3 and Figure 4, is incomplete. From a few thousand publications describing various phage display screens (1990-2010), > 50% did not report sequence abundances explicitly, ~ 30% of the articles (~200 out of 600 publications for Ph.D.-12 libraries) sequenced < 15 clones and only ~15% report sequences for 15 or more clones. Additionally, standard search engines (PubMed or ISI) are ill-suited for searching the phage display literature: they often do not contain information about the sequences nor the library type. The survey was facilitated by a database of phage sequences (MimoDB) generated by Jian Huang and co-workers (http://immunet.cn/mimodb) [49]. This database, however, provides partial information about sequence results only (e.g., binding affinities of sequences in Figure 3 and Figure 4 were not documented).

## 5. The Relative Abundances of Ligands in the Library Are Not Correlated to Their Binding Strength

**Figure 5:**
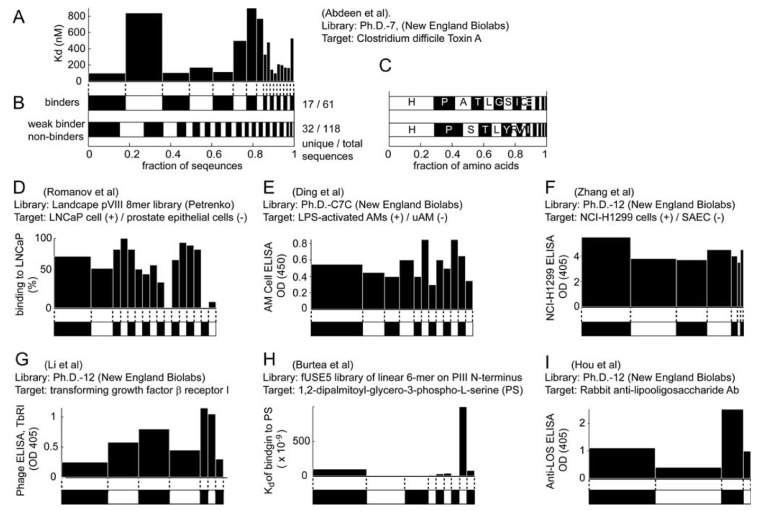
There is no correlation between the abundance of binding clones and their binding ability. **(A-C) **This example is from Feig and co-workers, who identified peptides that bind to *Clostridium difficile* toxins. After 4 rounds of panning, 61 clones were identified as binders and 118 clones had weak (or no) binding affinity [50]. **(A) **The K_d_ of the clones that bind. The horizontal position of each bar corresponds to the binder clone shown in the top stacked bar in **(B)**. The width of the bar in A and B indicates the abundance of each clone. The binding affinity of each clone and its abundance in the sub-library are not correlated. **(B)** The distribution of the diversity in sub-populations of binders and non-binders are similar (both have several highly abundant clones). **(C)** Amino acid abundances are similar. **(D-I)** Results from other screens in the literature have the same trend as those in A: binding ability of the phage clones and their abundances are not correlated [53,54,55,56,57,58].

If a decrease in the library diversity is primarily due to the specific binding of ligands to the target, the distribution of ligands in the final library should correlate with the binding ability of the phage. Such correlation cannot be drawn, however, based on results from the literature. As example, we used information from a screen performed by Andrew Feig and co-workers [50] because it contains a detailed analysis of the sequences of 179 clones and the K_d_ value for each binding clone. Of the 179 clones, 118 were weak binders or non-binders and 61 were binders (confirmed in follow-up assays). This study, summarized in Figure 5, demonstrates that: (1) the K_d_ of phage clones that present a given peptide sequence cannot be correlated to the abundance of this clone within the 179 clones sequenced (Figure 5A). (2) The distribution of the abundance of binding and non-binding sub-populations is similar (Figure 5B). (3) The amino acid distribution in these two populations is also similar (Figure 5C). Interestingly, the abundance of proline is observed in > 50% of the screens, as shown in Figure 4, and in the original, unselected libraries [24]. Pro is generally abundant in protein-protein interaction sites [51,52], but its abundance in phage-displayed peptides can also originate from the specifics of the phage secretion mechanism [24].

All three observations above suggest that there is the enrichment of specific binding clones and the elimination of other binding clones that were independent of their binding ability. The results in Figure 5A-C are representative of trends usually seen in the literature. The results in Figure 5D-I summarize the findings from different libraries and different targets [53,54,55,56,57,58]. They illustrate that the abundance of the binding clones is not correlated with their binding ability. Collecting a comprehensive set from the literature, however, was difficult because most reports sequenced only a small numbers of clones, performed qualitative “yes/no”-assays, or characterized a small number of clones.

## 6. Subtle Differences in Growth Rate Yield Drastic Differences in Clone Abundances after Rounds of Amplifications

**Figure 6:**
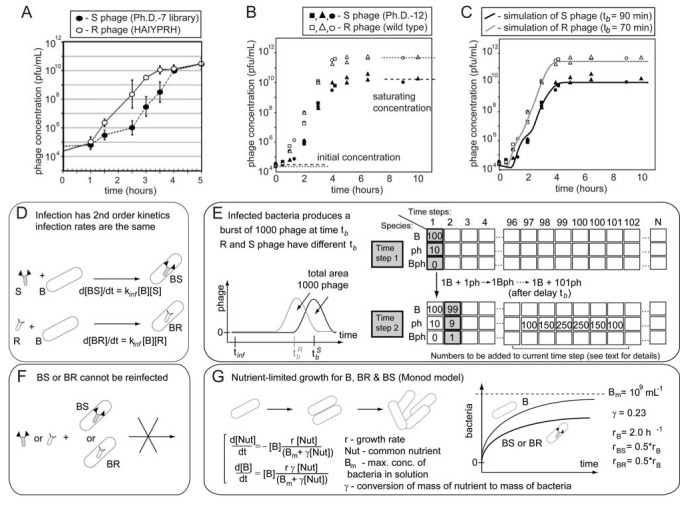
The kinetics of phage growth and its effects on the diversity of the library. **(A)** HAIYPRH-displaying phage amplifies faster than parent Ph.D.-7 library. In separate solutions, Ph.D.-7 library amplifies more slowly, but eventually reaches the same saturating concentration. **(B)** Competition of wild-type insert-free phage (R) and phages from Ph.D.-12 library (S) when these phage are amplified in the same solution. **(C) **Results from B overlaid with results from simulation of the competition between two clones. **(D-G)** A detailed description of the simulation: **(D)** Infection of phage (R or S) and bacteria (B) is a second order kinetic process with an infection rate constant k*_inf_* which produces infected bacteria (BR or BS). **(E)** BR or BS generate a burst of 1000 copies of R or S. Burst time follows a normal distribution. Average burst time is the only parameter that distinguishes R and S phage. **(F)** Infection by R or S converts bacteria B to BR or BS species that cannot be re-infected. **(G)** Bacteria grow via symmetric division according to substrate-limited Monod model. Growth rate of infected bacteria (BR or BS) is 2x slower than B. (**A** - adapted from Brammer *et al.* [39]; B – reproduced from Derda *et al.* [66] with permission).

The biological reasons for growth advantage have been discussed in several reports [36] and include: the binding to pili, the use of rare codons [37], interference with packing or infection [22,24], and rare mutations in the regulatory regions of phage genes [38,39]. These effects are usually small for the display of peptides on pIII proteins and for the display of short (<8-mer) peptides on protein pVIII [59,60,61]. In general, libraries of peptides displayed on pVIII are more prone to loss of sequence diversity than those displayed on pIII [22,25,62] but these problems can be mitigated by modification of pVIII proteins [63,64]*.* Phage display technology is very successful because the modification of the phage coat proteins has minor effects on the rate of production of phage [5,7,65]. Nevertheless, we demonstrate that even small differences in growth rate can have important consequences in the distribution of phage that display different peptides after amplification. Figure 6 shows two well-characterized examples using phage that display the 7-mer HAIYPRH-sequence and contain a mutation in the regulatory region of gene pII (Figure 6A) [39], and M13 phage with the wild type (wt) M13 genome (Figure 6B) [66]. For example, a wt (rapid or R) phage produces a burst of phage ~70 minutes after infection whereas a library (slow or S) phage requires ~90 minutes (Figure 6B) [66]. This difference of 40% between R and S phage was sufficient for the R (wt) phage to take over the population— the R/S ratio reached 300:1 after five hours of growth—that started with a 1:1 mixture of wt and library phage (Figure 6B). HAIYPRH-phage also rapidly takes over a population of library phage [39].

Growth advantages of different clones do not originate from differences in the total number of phage produced per bacteria. Library, wt, or HAIYPRH-phage reach the same saturation density when amplified in separate solutions (Figure 6A). Rather, growth advantages result from small differences in the growth rate of the different phage clones, and the exponential growth of phage. Infection produces 1000 copies of phage from a single bacterium and the number of phage grows as 1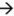 10^3^ 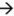 10^6^ 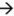 10^9^ upon serial infection in an excess of uninfected bacteria. We used a discrete-step model of phage growth to visualize how small differences in growth rate lead to large differences in the number of phage produced after multiple cycles of re-infection. The model used four parameters: (1) infection rate was described using a simple 2^nd^ order kinetics with respect to concentration of phage and bacteria (Figure 6D); (2) delayed burst of 1,000 phage particles (Figure 6E) [36]; (3) no re-infection of bacteria that were already infected (Figure 6F) [67]; and (4) substrate-limited growth for infected and non-infected bacteria (Figure 6G) [36,68]. The model fits well with the data in Figure 6B and we can therefore attribute the 1:1 to 1:300 increase in R/S ratio to ~30% difference in growth rates of R and S phage (Figure 6C).

**Figure 7:**
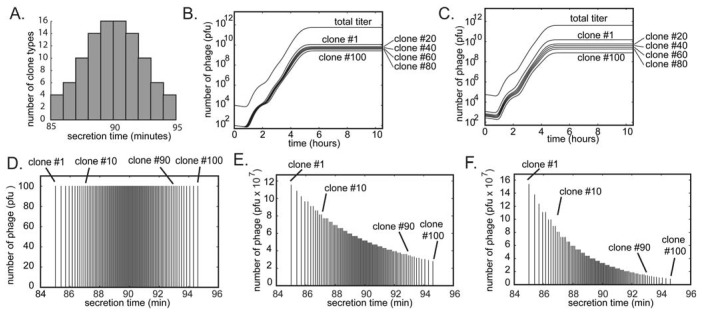
A simulation of the growth of a library containing 100 different clones. (**A **and **D**) The initial population contains equal concentrations of the clones (numbered #1 through #100). The clones differ only in the time they are produced by the bacteria (average burst time is slowest for #1 and fastest for #100). We approximate the burst time of different clones in the population to follow a Gaussian distribution (*i.e.*, the abundance of the fast and slow growers is low; most clones have an average growth rate). (**B**) Amplification of mixture of 100 copies of each clone using 10^8^ bacteria (see Figure 6D-G for details of the amplification). In amplification from 10^4^ pfu/mL to 10^12^ pfu/mL, the ratio of clone #1 (fastest) to clone #100 (slowest) reaches 5:1 (**E**). (**C**) Dilution of the amplification result from B to ~10^4^ and re-amplification to 10^12^ further skews the distribution of the clones (**F**).

We used the same model to describe the growth competition in a library containing 100 different clones that differ only in their secretion time (Figure 7). The distribution of secretion time of the clones was assumed to be normal (Figure 7A,D). The fastest clone #1 and the slowest clone #100 had secretion time of 85 and 95 minutes, respectively (Figure 7D). Starting from a population of clones with equal abundance, amplification produces a population of phage in which the ratio of clone #1 to clone #100 is 5:1 (Figure 7B,E). This ratio reaches 15:1 after dilution and re-amplification of the library (Figure 7C,E). This model confirms that as little as a 10% difference in growth rate among phage clones can be rapidly amplified to yield distributions of clones similar to those observed in real screens. 

The model we described in Figure 6 and Figure 7 does not provide the most precise description of all stages of life cycle of the phage. It shows, however, that a simple model that accounts for one difference in life cycle between phage clones—the rate of secretion—can be sufficient to explain the origin of large differences in concentration of the clones after amplification (Figure 6C). 

We have chosen secretion rate as a variable. The same model can be used to predict what differences in concentrations can arise from differences in infection rate, or a combination of infection rate, secretion rate, and other parameters. The accurate multi-parametric simulation of phage competition, however, will require extensive support from the experiments that measure all the relevant parameters (e.g., secretion rate, infection rate, *etc.*).

## 7. Relationships between Panning and Amplification

**Figure 8:**
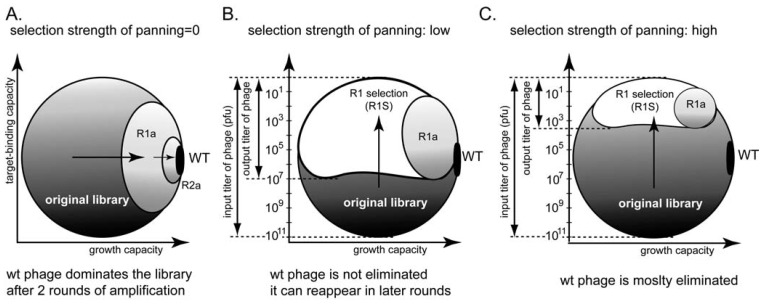
**(A-C)** Phage phase diagram illustrating that increasing the strength of the selection can minimize amplification of non-specific ligands.

Tuning the stringency of panning can be used to minimize the selection of non-specific ligands. For example, the ratio of input and output clones can be used to indicate different panning stringencies (Figure 8B,C). In the absence of panning, the screen yields one wild type clone that amplifies the fastest (Figure 8A) [66]. Increasing the strength of the selection can avoid selection of non-specific fast-amplifying clones (Figure 8C). It cannot, however, mitigate the competition among binding clones.

There are indications in the literature that a phage that displays peptides with a β-turn structure on pIII protein amplify faster, whereas those that display α-helical peptides amplify slower [24,69]. Panning for a target that binds α-helical peptides will select for slower growing clones; amplification, which selects for fast growing clones, would inevitably interfere with panning. Panning against a target that preferentially recognizes β-turn peptides, thus, simultaneously enriches for faster-growing clones. Direct evidence for this prediction, has not been demonstrated for the pIII display system, but selection of peptides of specific structure is known in the pVIII display system [22].

## 8. How Many Binding Ligands Are Lost in the Screen?

The phage diagram (Figure 1D-G) predicts that phage-display selection loses the majority of the binding ligands originally present in the library after several rounds of amplification. This loss can be quantified by estimating the number of potential ligands in the library [24,70]. For example, if the target recognizes a stretch of five amino acids, simple calculations show that a 5-mer binding motif occurs in within a 12-mer approximately 10^10^ times [71]. In a library of 12-mers (4.1 × 10^15^ unique sequences), 1 sequence in every 400,000 sequences is a frame-shifted binder. If the frame shift does not change the binding ability of the peptide, then a library with 10^9^ random peptides can contain ~10^3^ binding clones that present ligands of similar affinities. If the target has 1,000s of binding sites (e.g., a cell), the number of identified ligands should scale up further [72]. 

Peptide-based ligands can also contain permissive mutations in the recognition site that do not perturb binding. An estimation of the possible number of the permissive mutants can be based on the assumption that a specific class of amino acids (charged, hydrophilic, non-polar) at the specific position are required for binding. This assumption, however, is not general: Sidhu and co-workers, for example, demonstrated that the recognition of many targets can be achieved only by a combination of two amino acids (Tyr and Ser) located on a scaffold of defined shape (such as, the binding site of the antibody) [73,74]. Recognition of RNA/DNA aptamers [75,76] or molecular imprinted polymers [77] also suggests that the shape of the molecule can provide a rich recognition repertoire with very limited side-chain repertoire. The plasticity of molecular recognition and the current lack of understanding of molecular recognition in water make it difficult to predict the number of permissive mutants. It is safe to assume, however, that permissive mutations can increase the number of potential binders by 1-2 orders of magnitude. 

Panning alone cannot provide the selective pressure for enriching one peptide from thousands of other binders with similar affinities. Convergence to a few clones should never happen in an ideal situation where panning is the only selective pressure; this convergence, however, happens in 90% of the literature reports (Figure 3, Figure 4). 

## 9. Mitigating Amplification-Induced Convergence: Screens without Amplification

If amplification of a library leads to loss of diversity, the simplest strategy to bypass this problem is to skip the amplification steps altogether. This method is generally avoided because the ratio of binding clones to non-binding clones is believed to be lower. The method, however, has been used successfully in several instances. 

William and Sharon conducted a single round of panning to identify ligands from Fab-displaying phage libraries that bind to colorectal cancer cells [78]. 50-90% of the clones isolated from this single round were identified as binders to colorectal cancer cells in a follow-up ELISA. In a similar study, Sharon and co-workers performed one round of panning against *Cryptosporidium parvum* glycoproteins to identify a population of phage that contained 50-70% of active clones [79]. We have conducted an amplification-free panning experiment to isolate a population of ~10^5^ clones that bind to the surface of pluropotent cells [23]. From this population, using ELISA and arrays of peptides [80,81], we identified the binding clones (six out of 500 tested sequences) from which two supported long-term self-renewal of human ES cells [23].

Arap, Pasqualini and co-workers used a single round of panning to identify peptides that bind to tissues, or tissue-specific vasculature in a brain-dead human patient. Only one round of panning was used originally because isolation of panning targets—surgical removal of multiple vital tissues—precluded panning in the same human patient [82]. On the other hand, biopsy in non-vital sites (e.g., a tumor) can be performed multiple times; it is thus possible to conduct repetitive rounds of phage panning in the mouse [83] or human organism [84] (reviewed in [85]).

Smith and co-workers used a single round of panning to identify peptide substrates for proteases [86]. A fUSE5 phage vector was used to display a random hexamer and a FLAG sequence. After incubation with proteases, the phage that display peptides cleaved by proteases are FLAG-free, and can be easily isolated. Sequencing of 86 clones after one round of panning yielded 86 different peptides from which only six had no detectable substrate activity for the target proteases. One round was shown to yield results similar to three-rounds of panning [86].

Removing library amplification step from the screen eliminates one of the advantages of phage display. Nevertheless, even without re-amplification, screening of phage libraries has several advantages over screening of non-encoded libraries. Small molecules which are not displayed on phage cannot be screened as a complex mixture; each molecule has to be present at large quantities for subsequent identification. For example, 10^6^-10^8^ molecules are required for mass-spectrometry-based identification, whereas even one particle of phage can be easily isolated and characterized

## 10. Mitigating Amplification-Induced Convergence: Amplification in Isolated Compartments

Previous sections demonstrate that phage competition occurs due to different rates of production and not due to differences in the total numbers of phage produced (*i.e.,* phage with different secretion kinetics produce an equal number of clones). For example, both Ph.D.-12 library phage and wt phage produce similar number of clones (~10^13^) when amplified in separate solutions [66]. HAYPRI-phage and library phage also reach identical final number of clones when they amplify in separate compartments (Figure 6A) [39]. These observations suggest that uniform amplification—one that does not enrich any clones in the library and preserves their ratio—can be achieved by: (1) isolating clones from one another; (2) supplying each clone with an equal number of bacteria, (3) allowing the amplification process to go to completion (*i.e.,* allowing all bacteria to be infected by phage). 

### 10.1. Agarose plates as isolated compartments

**Figure 9:**
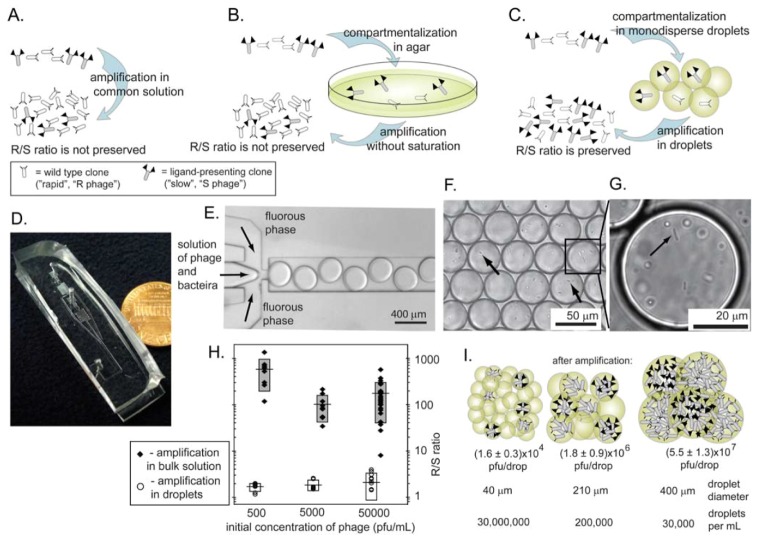
Amplification methods that prevent competition between the phage. **(A) **Rapid R and slow S clones compete in the same solution. The R/S ratio is not preserved. **(B)** R and S are isolated in agar, but they do not amplify to completion. The R/S ratio is not preserved. **(C)** Amplification in monodisperse droplets isolates each phage to its own droplet; amplification to saturation preserves R/S ratio. **(D)** Photograph of the microfluidic device we used to isolate phage in separate monodisperse droplets. **(E)** Optical micrograph of the microfluidic device that generates droplets containing bacteria and phage in LB media suspended in a perfluorinated solvent as the carrier fluid. **(F)** Droplets contain bacteria (arrows) and phage. We generated these drops from a solution containing phage with an initial concentration such that each drop of a specific volume contains one or zero particles of phage. **(G)** Dividing bacteria inside the droplet. **(H)** Comparison of the amplification of a mixture of R and S phage (see Figure 6) in bulk solution or in the droplets. The R/S ratio is preserved in droplets and it increases by 100 to 300-fold in bulk solution. **(F)** The size of the droplets is important; the number of phage generated per droplet is proportional to the size of the droplet. Uniform amplification, thus, can be obtained in monodisperse droplets and not polydisperse emulsions. Reproduced from Derda *et al.* [66] with permission.

One method for isolating phage is the growth of libraries as sub-confluent plaques in an agar overlay that contains excess of bacteria. Amplification in agar was used in several reports to produce and amplify phage displayed libraries [7,28,87,88]. The method was also commonly used in phage-based screens of DNA libraries that used bacteriophage λ [89]. Amplification in an agar overlay satisfies two of the three criteria above: (1) isolation and (2) an equal amount of bacteria. Phage clones in plaques in agar, however, grow continuously and never reach “true” saturation in which phage infects all of the available bacteria. Simple inspection of phage plaques reveals that the plaque sizes are not uniform (*i.e.*, growth rate in the individual plaques is different). Amplification by isolation in agar is reminiscent of incomplete amplification in Figure 6A; (e.g., ratio of HAIYPRH-phage to library phage in Figure 6 is non-uniform at any time before saturation at ~5 hours). Indeed, a comparison of the amplification in a mixture of rapidly-growing phage (M13mp8) and slow-growing phage (display of 38-amino acid sequence on M13mp18) demonstrates that plate amplification provided no significant advantage over liquid culture. Both amplification methods exhibit similar bias resulting from competition amongst clones (Figure 9A-B) [87]. Amplification of phage in agar overlays is also experimentally inconvenient. For example, ~10 cm^2^ of agar is required to isolate 500 clones. Amplification of a typical 10^5^-10^6^ output from the phage library, thus, requires agar trays of 2,000-20,000 cm^2^ (44 × 44 cm and 140 × 140 cm respectively).

### 10.2. Monodisperse droplets as isolated compartments

We have demonstrated, recently, that true uniform amplification of phage libraries can be achieved in monodisperse droplets formed in a microfluidics channel (Figure 9C-I) [66]. Monodisperse droplets satisfy all of the criteria outlined above: the drops isolate the phage clones; each compartment is identical in size and contain a similar number of bacteria that grow to nearly identical densities. Figure 9H compares the amplification of identical libraries in bulk solutions and in droplets. A 1:1 mixture of rapid-growing (R) and slow-growing (S) phage amplified uniformly in monodisperse compartments (Figure 9H). The original 1:1 ratio was preserved after amplification. Those that amplified in bulk solutions always yielded >300:1 R/S ratio after amplification. We also demonstrated explicitly that droplets must have uniform size in order to achieve uniform amplification (Figure 9I). The number of phage produced per droplet was proportional to the size of the droplet. Since the volume of droplets in polydisperse emulsions (e.g., those generated by vortex-mixing of oil and water) could vary > 10-100 fold [90], amplification of phage libraries in these droplets cannot be uniform.

The use of monodisperse droplets for the uniform amplification of phage was enabled by a series of advances in microfluidics technology: (1) microfluidic flow-focusing devices (MFFD) [91,92], T-junctions [93], and other geometries can generate droplets of <1% polydispersity at rates >10,000 droplets per second. This speed allows the production of a necessary amount of droplets in a short amount of time (e.g., 10^6^ in ~30 min). A library of phage and bacteria could, therefore, be mixed directly in LB media, and then encapsulated into separate droplets before the first burst of phage production has occurred (within 30–45 min) [66]. (2) Perfluorinated liquids were used as a carrier fluid for the droplets containing phage and bacteria. Perfluorinated liquids are highly permeable to oxygen, thus the growth of bacteria in droplets was not limited by oxygen [94]. (3) The development of a biocompatible fluoro-surfactant by Weitz and co-workers allowed the stabilization of droplets in perfluorinated liquids [95]. Emulsions containing phage and bacteria could be rocked in a Petri dish for many hours without causing the drops to coalesce. In addition, there was no transfer of bacteria or phage among these droplets [66]. Without a suitable surfactant, droplets have to be confined to the surface or isolated inside a long tubing to prevent coalescence during culture [96,97].

## 11. Indirect Mitigation of Amplification-Induced Convergence Using Bioinformatics Analysis

Libraries of phage-displayed peptides are limited in their diversity even before selection because they are secreted by bacteria. Rodi, Soares, and Makowski analyzed the diversity of the several libraries and demonstrated that the first secretion step makes certain types of sequences (e.g., proline-containing) more abundant than other sequences (e.g., cysteine-containing). From analysis of abundant sequences, Rodi and Makowski developed a method that calculated the probability (p) of finding a specific sequences in the original library [98]. They hypothesized that peptides of low information, or -ln(p) are common in the library, and are more likely to be present in the screen due to growth advantage. Those with high information are more likely to be selected due to panning [24]. Analysis of the information content of the peptide, hence, can be used to identify phage clones that were selected due to growth preferences [88,99].

## 12. Indirect Mitigation of Amplification-Induced Convergence Using Databases

This issue of the journal contains a review of the bioinformatics tools and phage databases [100]. Databases of phage sequencing results can be used to search for peptides that are commonly identified in peptide screens and thus identify ligands that are selected due to growth advantage [101]. Similar searches have led to the discovery of a phage that has mutation in the ribosome binding site (RBS), which equips these clones with a growth advantage [39]. 

Databases of sequences based on published results serve a useful purpose, but they can miss a lot of useful information, which is not reported in the publications. Bioinformatics databases are usually updated and supervised by a single user or group of users and it usually has fixed entry. We propose that leveraging both open-source and open-access along with semantic web technology can build a much more useful database than those currently. Ideally, the database can be maintained by individual users and contain auxiliary data like the sequencing results that are traditionally not reported in the publications. Information concerning the ongoing development of this project can be found at www.phagewiki.org. 

## 13. Loss of Diversity in Other Display Systems

The problem outlined in this review is not unique to peptide libraries on filamentous phage. Loss of diversity during amplification in an environment with shared recourses occurs for any replicating species. Joyce and Breaker demonstrated that simple re-amplification of RNA libraries in one solution selects “parasitic sequences” that amplify faster than the rest of the library [102]. Recently Zimmerman *et al.* investigated how rounds of amplification can influence the diversity of libraries of RNA aptamers using massive parallel sequencing [48]. Their results mirror many observations mentioned in our review. For example, the authors reported that “SELEX favors structurally unstable sequences in general, independent of the positive selection”. Interestingly, SELEX did not select for specific sequence or specific nucleotide content; still, it enriched for sub-classes of sequences that have lower structural stability (and thus, potentially, amplified faster) [48,103]. Any technique that uses DNA or RNA as an information carrier uses DNA amplification in a common solution—DNA display [104,105], RNA display [106], or ribosome display [107,108]—will suffer from this competition. This problem, however, can be solved by the separation of competing species into droplet-based compartments [66,109,110].

We note that compartmentalization of phage may not always result in uniform amplification. For example, mutations in a phage coat protein can decrease the number of produced phage from 10^13^ pfu/mL to 10^10^ pfu/mL [17,18]. In this case, compartmentalization will still lead to the loss in diversity. Concurrent use of orthogonal display systems—lytic phage T7 [111], or λ [89], chemically-resistant bacterial spores [112], physiologically benign *S. carnosus* [113,114], or yeast [115], and other systems [116,117]—can be used to identify a population of binding peptides that have growth disadvantages in libraries displayed on filamentous phage. 

## 14. Outlook

Elimination of the majority of possible binders and retaining only a few discards a lot of useful information. The undesired loss in target-binding clones was convenient in the past, when researchers could only isolate and characterize a small sub-population of phage clones due to practical and economic inconveniences. The loss of most binding clones, in some ways, made phage display practical in the times when DNA sequencing was slow and expensive. Enrichment of a few clones in a population of <20 clones was often used an indicator of the selection success. For targets with a single binding site, loss of extra binding ligands might not appear as a problem: researchers are usually interested in the “one ligand that works”. Even if given hundreds of peptides with identical affinities, it is not uncommon that only one would be picked for follow-up studies. Unfortunately, for a target that has multiple binding sites (e.g., cells), competition between binding clones makes it impossible to identify multiple ligands that bind to distinct sites on these targets. Clones that contain binding peptides compete during amplification and the majority of useful binders are eliminated from the screen.

Phage display is no longer limited by sequencing constrains. In the past 5-10 years, sequencing of large number of DNA sequences has become routine [118]. Commercial technologies like Illumina sequencing [119] or Polonator [120] make it possible to sequences hundreds of millions of short <100 bp DNA fragments. One example of deep-sequencing of phage (~100,000 clones using 454 technology [121]) has been reported by Pasqualini and Arap and co-workers [122]. The number of diverse sequences obtained from medium-scale 454 sequencing suggests that original libraries can be completely covered by large-scale sequencing. 

The elimination of competition between binding clones and large-scale sequencing of phage libraries will enable: (1) a prediction of affinity from the abundance of the clones. (2) a conclusion about the motifs that are absent from the results: motifs which are not enriched do not bind. Information about sequences that do not work can provide complementary information for structure-activity relationship (SAR) in addition to SAR based on binding sequences only. (3) the use of phage as a tool in forward chemical genetics [123,124]. A panning of peptide libraries against a cell can yield ligands for many cell-surface receptors. Exposing cells to these peptides identifies those that yield a desired phenotype (e.g., the inhibition of stem cell differentiation) [23,125]. The identification of the cognate receptors can lead to the discovery of the mechanism for regulation of biological processes (e.g., stem cells differentiation). This approach can sample a much higher number of binding ligands than traditional chemical genetic approaches (limited to 10^3^-10^4^ compounds) [126,127]. Its success, however, depends critically on the ability to identify ligands for all receptors on the surface of the cell. It is possible only if the binding ligands do not compete with one another during selection.
